# Association Between Systemic Inflammation and Malnutrition With Survival in Patients With Cancer Sarcopenia—A Prospective Multicenter Study

**DOI:** 10.3389/fnut.2021.811288

**Published:** 2022-02-07

**Authors:** Guo-Tian Ruan, Yi-Zhong Ge, Hai-Lun Xie, Chun-Lei Hu, Qi Zhang, Xi Zhang, Meng Tang, Meng-Meng Song, Xiao-Wei Zhang, Tong Liu, Xiang-Rui Li, Kang-Ping Zhang, Ming Yang, Qin-Qin Li, Yong-Bing Chen, Kai-Ying Yu, Marco Braga, Ming-Hua Cong, Kun-Hua Wang, Rocco Barazzoni, Han-Ping Shi

**Affiliations:** ^1^Department of Gastrointestinal Surgery/Department of Clinical Nutrition, Beijing Shijitan Hospital, Capital Medical University, Beijing, China; ^2^Key Laboratory of Cancer Food for Special Medical Purposes (FSMP) for State Market Regulation, Beijing, China; ^3^Department of Surgery, San Raffaele Hospital, Milan, Italy; ^4^Comprehensive Oncology Department, National Cancer Center/Cancer Hospital, Chinese Academy of Medical Sciences and Peking Union Medical College, Beijing, China; ^5^Department of Surgery, The First Affiliated Hospital of Kunming Medical University, Kunming, China; ^6^Department of Medical, Surgical and Health Sciences, University of Trieste, Trieste, Italy

**Keywords:** ALI, systemic inflammation, malnutrition, cancer sarcopenia, overall survival

## Abstract

**Objective:**

Systemic inflammation and malnutrition are correlated with cancer sarcopenia and have deleterious effects on oncological outcomes. However, the combined effect of inflammation and malnutrition in patients with cancer sarcopenia remains unclear.

**Methods:**

We prospectively collected information on 1,204 patients diagnosed with cancer sarcopenia. the mean (SD) age was 64.5 (11.4%) years, and 705 (58.60%) of the patients were male. The patients were categorized into the high advanced lung cancer inflammation index (ALI) group (≥18.39) and the low ALI group (<18.39) according to the optimal survival cut-off curve. We selected the optimal inflammation marker using the C-index, decision curve analysis (DCA), and a prognostic receiver operating characteristic curve. Univariate and multivariate survival analyses were performed to determine the prognostic value of the optimal inflammation indicator. We also analyzed the association between inflammation and malnutrition in patients with cancer.

**Results:**

The C-index, DCA, and prognostic area under the curve of ALI in patients with cancer sarcopenia were higher or better than those of neutrophil-lymphocyte ratio (NLR), prognostic nutritional index (PNI), systemic immune-inflammation index (SII), and *platelet-lymphocyte* ratio (PLR). The prognosis for patients in the low ALI group was worse than that of patients in the high ALI group [HR (95%CI) = 1.584 (1.280–1.959), *P* < 0.001]. When the ALI was divided into quartiles, we observed that decreased ALI scores strongly correlated with decreased overall survival (OS). Patients with both a low ALI and severe malnutrition (vs. patients with high ALI and well-nourished) had a 2.262-fold death risk (*P* < 0.001). Subgroup analysis showed a significant interactive association between the ALI and death risk in terms of TNM stage (*P* for interaction = 0.030).

**Conclusions:**

The inflammation indicator of the ALI was better than those of the NLR, PNI, SII, and PLR in patients with cancer sarcopenia. Inflammation combined with severe malnutrition has a nearly 3-fold death risk in patients with cancer sarcopenia, suggesting that reducing systemic inflammation, strengthening nutritional intervention, and improving skeletal muscle mass are necessary.

## Introduction

The European Working Group on Sarcopenia in Older People (EWGSOP) (1) and the Asian Working Group for Sarcopenia (AWGS) (2) have recommended that in the definition of skeletal sarcopenia, the loss of muscle strength and functional impairment should be increased on the basis of the loss of muscle mass. Cancer-related sarcopenia is considered part of cancer cachexia syndrome and is caused by a negative balance of protein and energy due to metabolic abnormalities and reduced food intake (3). Sarcopenia can cause contractile dysfunction, metabolic and endocrine abnormalities, and affect the systemic metabolism and immune and inflammatory responses (4).

Sarcopenia is a condition caused by systemic inflammation, commonly found in malignancy. As part of the tumor's systemic inflammatory response, pro-inflammatory cytokines and growth factors have a profound catabolic effect on the host's metabolism, leading to muscle failure (5). Low muscularity may lead to local muscle inflammation, and further to damage driving systemic inflammation (6). This inflammatory cycle, in turn, can enhance tumor aggressiveness or reduce response to treatment, impairing the transition to survival (7). Additionally, systemic inflammation is related to anorexia and insufficient nutrient intake, which in turn leads to accelerated loss of skeletal muscle and adipose tissue (4). Remarkably, cancer sarcopenia is an aspect of cancer-related malnutrition and is thought to have a negative impact on the survival of patients with cancer patients (8, 9). Accordingly, a low nutritional status is usually associated with sarcopenia. Early detection of malnourished patients and nutritional interventions is essential. The Patient-Generated Subjective Global Assessment (PG-SGA) nutrition evaluation tool is based on the SGA and is specifically developed for patients with cancer. The scored PG-SGA further develops the PG-SGA concept, which includes a numerical score and provides a global rating for good, moderate, or suspected malnutrition or severe malnutrition (10).

To our knowledge, no relevant study has investigated the combined association of the systemic inflammatory response and cancer malnutrition in patients with cancer sarcopenia survival. Systemic inflammatory response (SIR) markers, such as Serum C-reactive protein (CRP), hypoalbuminemia, absolute white blood cell count (WBC), and their components have been shown to play essential roles in the development and progression of cancer (11). At present, the predictive ability of inflammation-related cancer prognostic indexes such as the neutrophil-lymphocyte ratio (NLR), advanced lung cancer inflammation index (ALI), prognostic nutritional index (PNI), systemic immune-inflammation index (SII), and platelet-lymphocyte ratio (PLR) in patients with cancer sarcopenia is unknown. The purpose of this study was to identify an optimal inflammation indicator among these indicators and to investigate the combined prognostic effects of inflammation and malnutrition in patients with cancer sarcopenia.

## Materials and Methods

### Study Population

The Investigation on Nutrition Status and its Clinical Outcome of Common Cancers (“INSCOC”) was a prospective cohort gathered from multiple clinical centers for patients with cancer in China (June 2012 to December 2019). The inclusion criteria were: age ≥18 years, hospitalization ≥48 h, and pathological diagnosis of cancer. The protocol was approved by the local ethics committee of the participating clinical centers, and all patients provided signed informed consent (Registration Number: ChiCTR1800020329).

### Data Collection and Definitions

This study mainly included common population baseline characteristics, inflammation-related indicators, body measurements, laboratory examinations, and nutrition-related evaluation indicators. Body measurements were performed in strict accordance with the patient's admission with light inpatient clothing and socks in a relaxed state. Laboratory indicators were obtained without intervention before admission, and nutritional assessment was performed by specially trained professionals. Eleven major cancer types were included: lung, gastric, colorectal, esophageal, hepatobiliary, pancreatic, breast, uterine ovarian, nasopharyngeal, and urological cancer, and other cancer subtypes.

Body mass index (BMI, kg/m^2^) was calculated by dividing the weight by the square of the height. The BMI classification was based on Chinese standards. The included inflammation indexes included: the NLR (neutrophil count/ lymphocyte count), PLR (platelet count/lymphocyte count), PNI [10 × albumin (g/dl) + 0.005 × lymphocyte count], SII (platelet count × neutrophil count/lymphocyte count), ALI [BMI (kg/m^2^) × albumin (g/dl)/NLR]. The nutritional status of patients was assessed using the PG-SGA criteria, including patient self-evaluation and professional evaluation. According to the PGSGA score, the patients were classified into three different nutritional statuses: well-nourished (0–3), moderately malnourished (4–8), and severely malnourished (≥9).

### Assessment of Cancer Sarcopenia

According to the 2019 AWGS sarcopenia diagnosis consensus, the diagnosis of sarcopenia is based on a combination of a low appendicular skeletal muscle index (ASMI) and low muscle strength (handgrip strength, HGS) (12). For the HGS measurement, the handle was individually adjusted according to the size of the patient's hand. During the measurement, the surveyor guided or helped the patient to sit upright, with the arm resting on the armrest and the elbow bend 90°. Demonstrate the operation steps first and then instructed the patient to hold the handle with maximum strength within 3 s. The test was carried out thrice, and the maximum hand strength was recorded as the result. ASM was estimated using an equation that has been described and validated for the Chinese population: ASM = 0.193 × body weight + 0.107 × height (cm)−4.157 × sex−0.037 × age−2.631 (13). Bodyweight, height, and age were measured in kg, cm, and years, respectively. Male sex was coded as 1 and female sex as 2 (13–15). The ASM equation model is in good agreement with double X-ray absorptiometer measurements (adjusted *R*^2^ = 0.90, standard error of estimate = 1.63 kg) (13). After estimating the ASM values, ASMI was calculated as follows: SMI = ASM/height^2^ (m^2^) (14, 15).

The cut-off value that defined low muscle mass was based on the ASMI of the lowest 20% percentile in the study population (14, 15). The low ASMI classification criterion was: male <6.946 kg/m^2^ and female <5.421 kg/m^2^. The classification standard for low grip strength was male <28 kg and female <18 kg (12).

### Outcomes

The primary observational endpoint of this study was the patient's overall survival (OS), that is, the patient's survival time from the time of cancer diagnosis to the time of death, the time of withdrawal from the study, or the last follow-up time. A professional follow-up team conducted the clinical follow-up *via* telephone and outpatient or hospitalization records.

### Statistical Analysis

In this study, the inflammation indicators were divided into high- and low-groups, as calculated using log-rank statistics with R software to obtain the best survival cut-off value, namely high NLR (≥3.13) vs. low NLR (<3.13), high PLR (≥250.57) vs. low PLR (<250.57), high PNI (≥42.4) vs. low PNI (<42.4), high SII (≥968.33) vs. low SII (<968.33), and high ALI (≥18.39) vs. low ALI (<18.39) (Supplementary Figure S1). Additionally, the ALI score was stratified into quartiles based on baseline ALI score. Continuous variables are presented as the mean ± standard deviation (SD); the median (interquartile range) was used if necessary, and the unpaired Student's *t*-test was used for comparison between groups. Discontinuous variables are presented as percentiles (%), and comparisons between groups were performed using the chi-square test.

The selection of the best prognostic index was determined by using the prognostic receiver operator characteristic curve (ROC), decision curve analysis (DCA), and Harrell's concordance index (C-index). Pearson's correlations between the ALI and potential clinical parameters were computed. OS was calculated using the Kaplan-Meier method. To evaluate the risk ratios (HRs) and 95% confidence intervals (CIs) of OS, multivariate Cox survival regression analysis was performed using different adjustment models to reduce clinical deviation. Model 0: unadjusted; Model 1: adjusted for age, sex, and TNM stage; Model 2: adjusted for age, sex, radical resection, TNM stage, the European Organization for Research and Treatment of Cancer, Quality of Life Questionnaire-Core 30 (EORTC QLQ-C30), Karnofsky Performance Status (KPS), neoadjuvant chemoradiotherapy, post-operative chemoradiotherapy, lymphocytes, neutrophils, WBC, aspartate aminotransferase, alanine transaminase, serum albumin, comorbid disease (s), family history of cancer, tea consumption, alcohol consumption, smoking, platelet count, hemoglobin, total serum protein, PGSGA, nutritional intervention, 30-day mortality, HGS, and tumor types. The sensitivity analysis was performed by excluding patients who died within 6 months and those with TNM stage IV, respectively. We also constructed cube plots to estimate the relationship between the ALI and HRs of OS. Models were adjusted for model 2.

All statistical analyses were performed using the R platform (version 4.0.3, https://www.r-project.org/), and a two-tailed *P* < 0.05, was regarded statistically significant. The R packages we used in this study included: “survminer,” “survival,” “rms,” “foreign,” “timeROC,” and “ggplot2.”

## Results

### Baseline Characteristics

A total of 9,727 patients with cancer were included in the cohort study, of whom 1,204 patients were diagnosed with sarcopenia (Supplementary Figure S2). In the baseline data, the mean age of the patients was 64.5 ± 11.4 years and there were 705 male patients (58.60%). Among the main common cancer types, there were 239 (19.90%) cases of lung cancer, 245 (20.30%) of gastric cancer, 270 (22.40%) of colorectal cancer, and 145 (12.00%) of esophageal cancer. Additionally, 1,000 patients were diagnosed with malnutrition, including 398 (33.10%) cases of moderate malnutrition and 602 (50.00%) of severe malnutrition. However, only 328 (27.20%) patients received nutritional intervention (Table 1).

**Table 1:** Demographic and clinical characteristics.

	**Overall**
Characteristics	Patients (*n*, %)
	(*n* = 1,204)
Age, years, [mean (SD)]	64.51 (11.42)
**Sex**, ***n*** **(%)**	
Male	705 (58.60)
Female	499 (41.40)
**Sites of cancer**, ***n*** **(%)**	
Lung cancer, *n* (%)	239 (19.90)
Gastric cancer, *n* (%)	245 (20.30)
Colorectal cancer, *n* (%)	270 (22.40)
Esophageal cancer, *n* (%)	145 (12.00)
Hepatobiliary cancer, *n* (%)	31 (2.60)
Pancreatic cancer, *n* (%)	42 (3.50)
Breast cancer, *n* (%)	64 (5.30)
Utero ovarian cancer, *n* (%)	65 (5.40)
Nasopharyngeal cancer, *n* (%)	55 (4.60)
Urological cancer, *n* (%)	17 (1.40)
Other cancer subtypes, *n* (%)	31 (2.60)
**Comorbid disease(s), yes**, ***n*** **(%)**	
0	755 (62.70)
1	320 (26.60)
2	87 (7.20)
3 or more	42 (3.50)
Family history of cancer, yes, *n* (%)	159 (13.20)
Smoking, yes, *n* (%)	572 (47.50)
Alcohol consumption, yes, *n* (%)	242 (20.10)
Tea consumption, *n* (%)	280 (23.30)
BMI, kg/m^2^ [mean (SD)]	18.42 (1.78)
**TNM stage**, ***n*** **(%)**	
I	107 (8.90)
II	249 (20.70)
III	303 (25.20)
IV	545 (45.30)
Radical resection, yes, *n* (%)	347 (28.80)
Neoadjuvant chemoradiotherapy, yes, *n* (%)	46 (3.80)
Postoperative chemoradiotherapy, yes, *n* (%)	560 (46.50)
EORTC QLQ-C30	49.80 (9.280)
KPS [mean (SD)]	79.95 (17.39)
Serum total protein (g/L) [mean (SD)]	65.30 (8.19)
Serum albumin (g/L) [mean (SD)]	36.29 (5.78)
AST (U/L) [median (IQR)]	21.90 (17.00, 29.93)
ALT (U/L) [median (IQR)]	17.00 (11.18, 30.88)
Hemoglobin (g/L) [mean (SD)]	113.36 (21.20)
WBC ( × 10^9^/L) [mean (SD)]	7.05 (3.72)
Neutrophils ( × 10^9^/L) [mean (SD)]	4.92 (3.57)
Lymphocytes ( × 10^9^/L) [mean (SD)]	1.41 (0.87)
Platelet ( × 10^9^/L) [mean (SD)]	231.35 (97.95)
30-day death, yes, *n* (%)	32 (2.70)
**PGSGA**, ***n*** **(%)**	
Well-nourished	204 (16.90)
Moderately malnourished	398 (33.10)
Severely malnourished	602 (50.00)
Nutritional intervention, yes, *n* (%)	328 (27.20)
HGS [mean (SD)], (Kg)	16.55 (6.41)
ALI [median (IQR)]	21.26 (10.84, 32.26)
NLR [median (IQR)]	3.13 (1.85, 5.59)
PNI [median (IQR)]	43.50 (38.40, 48.30)
SII [median (IQR)]	679.00 (363.03, 1,132.47)
PLR [median (IQR)]	171.25 (114.96, 256.39)

*BMI, Body Mass Index; EORTC QLQ-C30, The European Organization for Research and Treatment of Cancer (EORTC), Quality of Life Questionnaire-Core 30 (QLQ-C30); KPS, Karnofsky Performance Status; AST, Aspartate Aminotransferase; ALT, Alanine Transaminase; WBC, White Blood Cells; ALI, Advanced Lung Cancer Inflammation Index; NLR, Neutrophil-Lymphocyte Ratio; PNI, Prognostic Nutritional Index; SII, Systemic Immune-Inflammation Index; PLR, Platelet-Lymphocyte Ratio; HGS, Hand grip strength; PGSGA, Patient-Generated Subjective Global Assessment*.

During the 43.7 months median follow-up period, with an estimated median OS of 25.7 months, we observed 572 deaths. We also observed the total mortality of this population from 1 to 5 years, namely, 36.4% (95% CI 60.9–66.4%) at 1 year, 48.7% (95% CI 48.4–54.3%) at 2 years, 54.6% (95% CI 42.4–48.5%) at 3 years, 57.1% (95% CI 39.9–46.2%) at 4 years, and 58.2% (95% CI 38.7–45.2%) at 5 years, amounting to a rate of 270 events per 1,000 patient-years.

### Comparison of Inflammation Indicators

**Figure 1:**
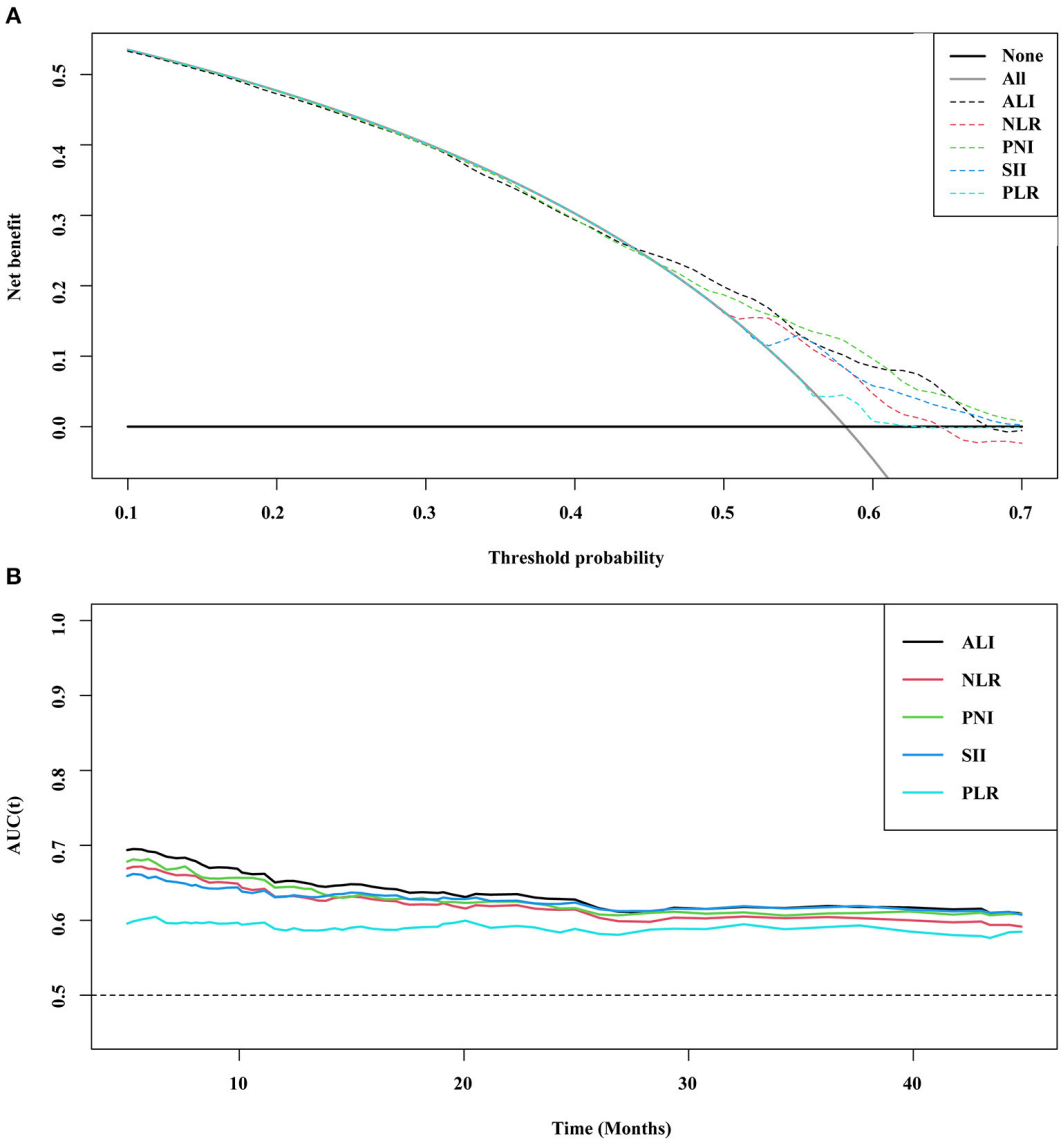
DCA and prognostic ROC of different inflammation markers **(A)** DCA; **(B)** ROC. ALI, Advanced Lung Cancer Inflammation Index; NLR, Neutrophil-Lymphocyte Ratio; PNI, Prognostic Nutritional Index; SII, Systemic Immune-Inflammation Index; PLR, Platelet-Lymphocyte Ratio; ROC, Receiver Operating Characteristic Curve; DCA, Decision Curve Analysis.

Five inflammatory indicators were analyzed and compared in terms of prognostic prediction and distinguishing ability in patients with cancer sarcopenia, namely the NLR, PLR, PNI, SII, and ALI. The C-index showed that the ALI [0.629 (0.606–0.652)] was superior to other inflammatory indexes [NLR (C-index 95%CI) = 0.614 (0.590–0.637), *P* < 0.001; PNI (C-index 95%CI) = 0.618 (0.594–0.642), *P* = 0.251; SII (C-index 95%CI) = 0.613 (0.5890.636), *P* = 0.013; and PLR (C-index 95%CI) = 0.575 (0.5500.599), *P* = 0.038], and the DCA curve suggested that the prognostic distinguishing ability and clinical application value of the ALI were superior to those of the other inflammatory indexes. The prognostic ROC curve indicated consistent results; that is, the area under the curve (AUC) of ALI was larger than that of other inflammation indicators (Figure 1).

### Distribution, Correlation, and Prognostic Analysis Based on the ALI

**Figure 2:**
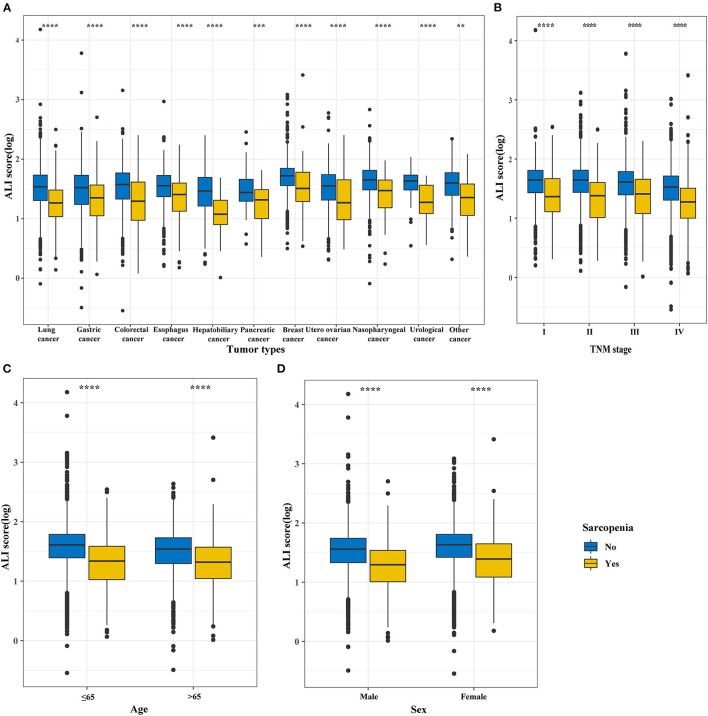
The distribution of the ALI in different groups based on sarcopenia subgroup and non-sarcopenia subgroup divisions. **(A)** Tumor types; **(B)** TNM stages; **(C)** Age; **(D)** Sex. ALI, Advanced Lung Cancer Inflammation Index.

Based on the total cohort (*n* = 9,727), we analyzed the distribution of the ALI in different cancer types, TNM stages, ages, and sexes, finding that the ALI scores of the patients with cancer sarcopenia were significantly lower than those of patients with non-cancer sarcopenia (all *P* < 0.001) (Figure 2).

Baseline data based on ALI stratification showed that sex, cancer type, BMI, TNM stage, EORTC QLQ-C30, KPS, total serum protein, serum albumin, hemoglobin, WBS, neutrophils, lymphocytes, platelets, 30-day mortality, PGSGA, and nutritional intervention were significantly different between the high and low ALI groups (Supplementary Table S1). We further analyzed the EORTC QLQ-C30 scores among the different ALI groups, and found that the functional status score and quality of life scores of patients with cancer sarcopenia in the low ALI group were significantly lower than those of corresponding patients in the high ALI group (all *P* < 0.001), but the symptom score of the low ALI group was significantly higher than that of the high ALI group (*P* < 0.001; Supplementary Table S2).

**Figure 3:**
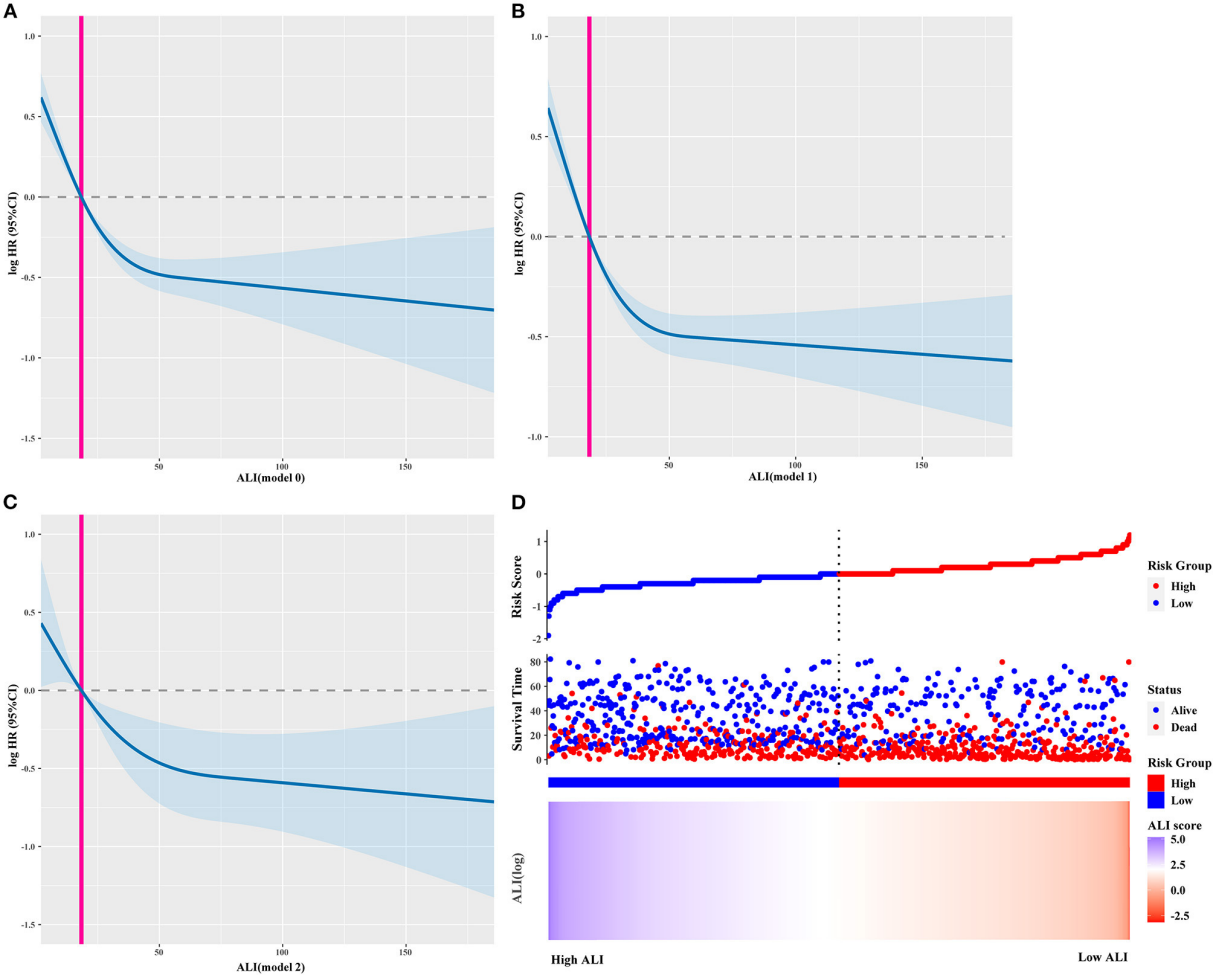
The restricted cubic spline curves and ALI risk model curve. **(A–C)** The restricted cubic spline curves of different adjusted models, **(A)** model 0: Unadjusted; Model 1: Adjusted for Age, Sex and TNM stage; Model 2: Adjusted for Age, Sex, Radical resection, TNM stage, EORTC QLQ-C30, KPS, Neoadjuvant chemoradiotherapy, Postoperative chemoradiotherapy, Lymphocytes, Neutrophils, WBC, AST, ALT, Serum albumin, Comorbid disease (s), Family history of cancer, Tea consumption, Alcohol consumption, Smoking, Platelet, Hemoglobin, Serum total protein, PGSGA, Nutritional intervention, 30-day mortality, HGS; **(D)** ALI risk model curve. ALI, Advanced Lung Cancer Inflammation Index; WBC, White Blood Cells; AST, Aspartate Aminotransferase; ALT, Alanine Transaminase; HGS, Hand grip strength; BMI, Body Mass Index; EORTC QLQ-C30, The European Organization for Research and Treatment of Cancer (EORTC), Quality of Life Questionnaire-Core 30 (QLQ-C30); PGSGA, Patient-Generated Subjective Global Assessment; KPS, Karnofsky Performance Status.

The calibration curve showed that the ALI had good predictive ability in patients with cancer sarcopenia at 1, 3, and 5-years (Supplementary Figure S3). The survival curve showed that the survival of patients with low ALI was worse than that of patients with high ALI (*P* < 0.0001; Supplementary Figure S4A). The restricted cubic spline curves showed that the HR of patients decreased with an increase in the ALI, showing an “L-shaped” linear relationship (Figures 3A–C). Similarly, as the ALI decreased, the risk of death increased (Figure 3D). Multivariate survival analysis showed that when the ALI was used as a continuous variable, the risk of death in patients decreased as the ALI increased [model 2: adjusted HR (95%CI) = 0.776 (0.562–1.072), *P* = 0.124]. When ALI was used as a binary variable, the prognosis of patients with low ALI was significantly worse than that of patients with high ALI [model 2: adjusted HR (95%CI) = 1.584 (1.280–1.959), *P* < 0.001]. When ALI was divided into quartiles, compared with the quartile 1 group (>37.94), the risk of death of patients in quartile 2–4 groups was significantly increased [model 2: *P* for trends <0.001; quartile 2 group (21.26–37.94): 1.330 (1.021–1.734), *P* = 0.035; quartile 3 group (10.84–21.26): 1.870 (1.423–2.458), *P* < 0.001; quartile 4 group (<10.84): 2.145 (1.511–3.044), *P* < 0.001] (Table 2). Sensitivity analysis was performed by excluding patients who died within 6 months and those whose TNM stage was IV. The results were consistent with the previously described findings (Supplementary Table S3). The prognostic analysis results of different tumor subgroups showed that a low ALI was associated with significantly worse prognosis in patients with colorectal cancer when compared with those patients with high ALI [model 2: adjusted HR (95%CI) = 2.347 (1.286–4.284), *P* = 0.005] (Supplementary Table S4).

**Table 2:** Univariate and multivariate analysis of the OS in patients with cancer sarcopenia.

**Variables**	**OS (model 0)**		**OS (model 1)**		**OS (model 2)**	
	**Crude HR (95%CI)**	**Crude *P***	**Adjusted HR (95%CI)**	**Adjusted *P***	**Adjusted HR (95%CI)**	**Adjusted *P***
**ALI**						
As continuous	0.679 (0.503–0.916)	0.011	0.755 (0.570–1.001)	0.051	0.776 (0.562–1.072)	0.124
**By cut-off**						
ALI ≥ 18.39	1		1		1	
ALI <18.39	2.063 (1.763–2.413)	<0.001	2.058 (1.757–2.412)	<0.001	1.584 (1.280–1.959)	<0.001
**By Interquartile**						
Q1 (37.94~)	1		1		1	
Q2 (21.26~37.94)	1.566 (1.215–2.019)	0.001	1.356 (1.051–1.750)	0.019	1.330 (1.021–1.734)	0.035
Q3 (10.84~21.26)	2.412 (1.896–3.068)	<0.001	2.088 (1.639–2.660)	<0.001	1.870 (1.423–2.458)	<0.001
Q4 (~10.84)	2.745 (2.163–3.484)	<0.001	2.749 (2.162–3.496)	<0.001	2.145 (1.511–3.044)	<0.001
*P* for trends		<0.001		<0.001		<0.001

*Model 0: Unadjusted; Model 1: Adjusted for Age, Sex and TNM stage; Model 2: Adjusted for Age, Sex, Radical resection, TNM stage, EORTC QLQ-C30, KPS, Neoadjuvant chemoradiotherapy, Postoperative chemoradiotherapy, Lymphocytes, Neutrophils, WBC, AST, ALT, Serum albumin, Comorbid disease(s), Family history of cancer, Tea consumption, Alcohol consumption, Smoking, Platelet, Hemoglobin, Serum total protein, PGSGA, Nutritional intervention, 30-day mortality, and tumor types, HGS. OS, Overall Survival; HR, Hazards Ratio; CI, Confidence Interval; BMI: Body Mass Index; EORTC QLQ-C30, The European Organization for Research and Treatment of Cancer (EORTC), Quality of Life Questionnaire-Core 30 (QLQ-C30); KPS, Karnofsky Performance Status; AST: Aspartate Aminotransferase; ALT: Alanine Transaminase; WBC: White Blood Cells; HGS: Hand grip strength; ALI: Advanced Lung Cancer Inflammation Index; PGSGA: Patient-Generated Subjective Global Assessment*.

### Combined Effect of the ALI and Malnutrition

**Figure 4:**
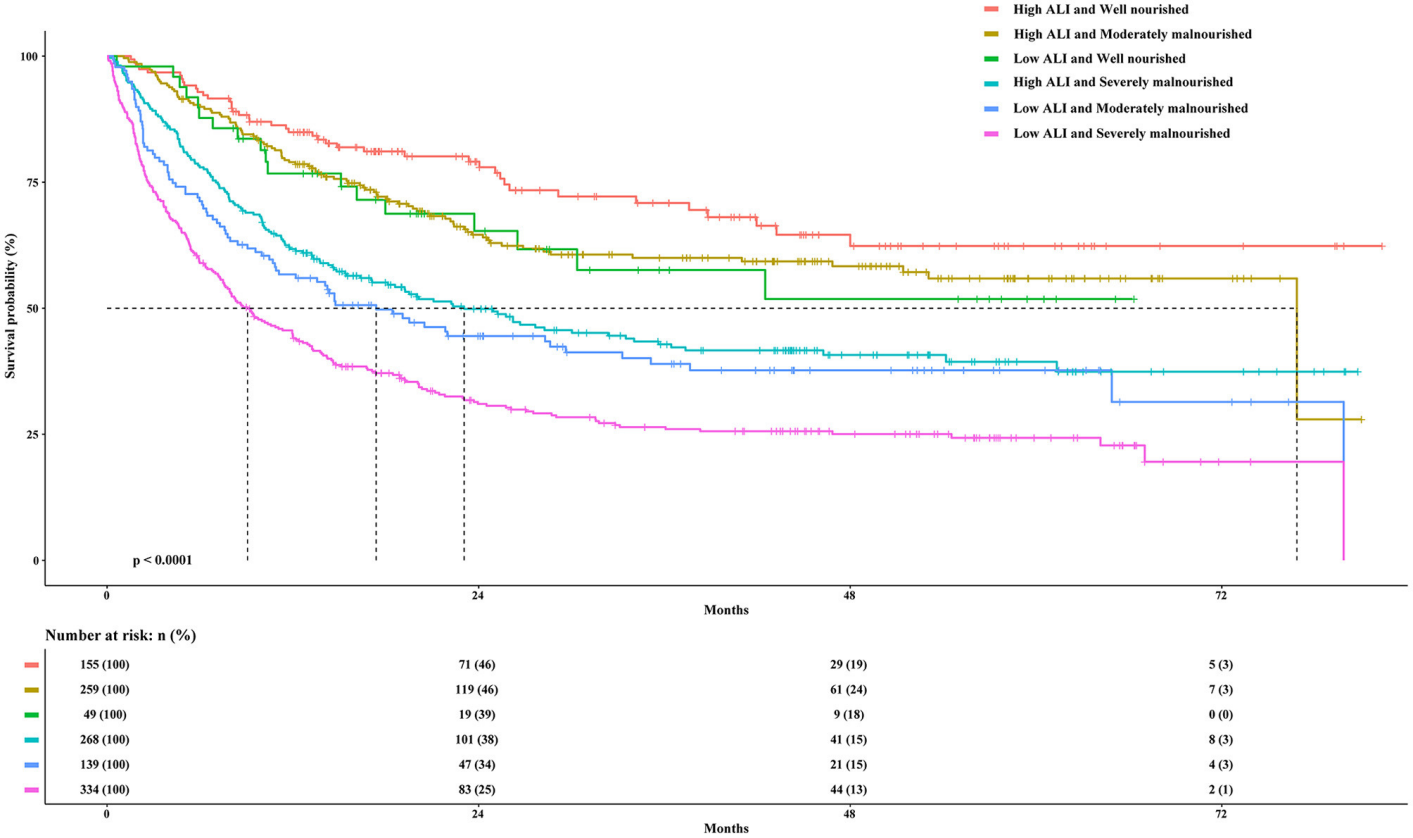
The Kaplan-Meier survival curves of the ALI combined with the PGSGA for the OS of patients with sarcopenia. ALI, Advanced Lung Cancer Inflammation Index; PGSGA, Patient-Generated Subjective Global Assessment; OS, overall survival.

First, we calculated the prognostic value of the PGSGA in patients with cancer sarcopenia (Supplementary Figure S4B). Univariate and multivariate survival analyses showed that compared with well-nourished patients, moderately malnourished patients (Adjusted HR = 1.207, 95%CI = 0.886–1.646, *P* = 0.233) and severely malnourished patients (Adjusted HR = 1.561, 95%CI = 1.148–2.123, *P* = 0.004) had a worse prognosis (Table 3; Figure 4). Additionally, we performed a combined survival analysis of the ALI and PGSGA in patients with cancer sarcopenia, and the results showed that compared with patients with high ALI and who were well-nourished, the risk of death in patients with low ALI who had severe malnutrition was 2.262-fold (95%CI = 1.527–3.351, *P* < 0.001) (Table 3).

**Table 3:** Combined effect survival analysis.

**Variables**	**OS**		OS^*^	
	**Crude HR (95%CI)**	**Crude *P***	**Adjusted HR (95%CI)**	**Adjusted *P***
**PGSGA^a^**				
Well-nourished	1		1	
Moderately malnourished	1.651 (1.233–2.211)	0.001	1.207 (0.886–1.646)	0.233
Severely malnourished	2.922 (2.226–3.835)	<0.001	1.561 (1.148–2.123)	0.004
**PGSGA and ALI** ^b^				
High ALI and Well-nourished	1		1	
High ALI and Moderately malnourished	1.408 (0.980–2.021)	0.064	1.081 (0.741–1.578)	0.687
High ALI and Severely malnourished	2.397 (1.701–3.377)	<0.001	1.448 (0.812–2.584)	0.210
Low ALI and Well-nourished	1.480 (0.852–2.571)	0.164	1.558 (1.079–2.250)	0.018
Low ALI and Moderately malnourished	2.837 (1.957–4.112)	<0.001	2.097 (1.383–3.178)	<0.001
Low ALI and Severely malnourished	4.118 (2.967–5.715)	<0.001	2.262 (1.527–3.351)	<0.001

*OS, Overall Survival; HR, Hazards Ratio; CI, Confidence Interval; BMI, Body Mass Index; EORTC QLQ-C30, The European Organization for Research and Treatment of Cancer (EORTC), Quality of Life Questionnaire-Core 30 (QLQ-C30); KPS, Karnofsky Performance Status; AST, Aspartate Aminotransferase; ALT, Alanine Transaminase; WBC, White Blood Cells; HGS, Hand grip strength; ALI, Advanced Lung Cancer Inflammation Index; PGSGA, Patient-Generated Subjective Global Assessment*.

* *Adjusted model*.

b *Adjusted for Age, Sex, Radical resection, TNM stage, EORTC QLQ-C30, KPS, Neoadjuvant chemoradiotherapy, Postoperative chemoradiotherapy, Lymphocytes, Neutrophils, WBC, AST, ALT, Serum albumin, Comorbid disease(s), Family history of cancer, Tea consumption, Alcohol consumption, Smoking, Platelet, Hemoglobin, Serum total protein, Nutritional intervention, 30-day mortality, HGS, and tumor types*.

b *Adjusted for Age, Sex, Radical resection, TNM stage, EORTC QLQ-C30, KPS, Neoadjuvant chemoradiotherapy, Postoperative chemoradiotherapy, Lymphocytes, Neutrophils, WBC, AST, ALT, Serum albumin, Comorbid disease(s), Family history of cancer, Tea consumption, Alcohol consumption, Smoking, Platelet, Hemoglobin, Serum total protein, Nutritional intervention, 30-day mortality, HGS, and tumor types*.

### Subgroup Analysis

**Figure 5:**
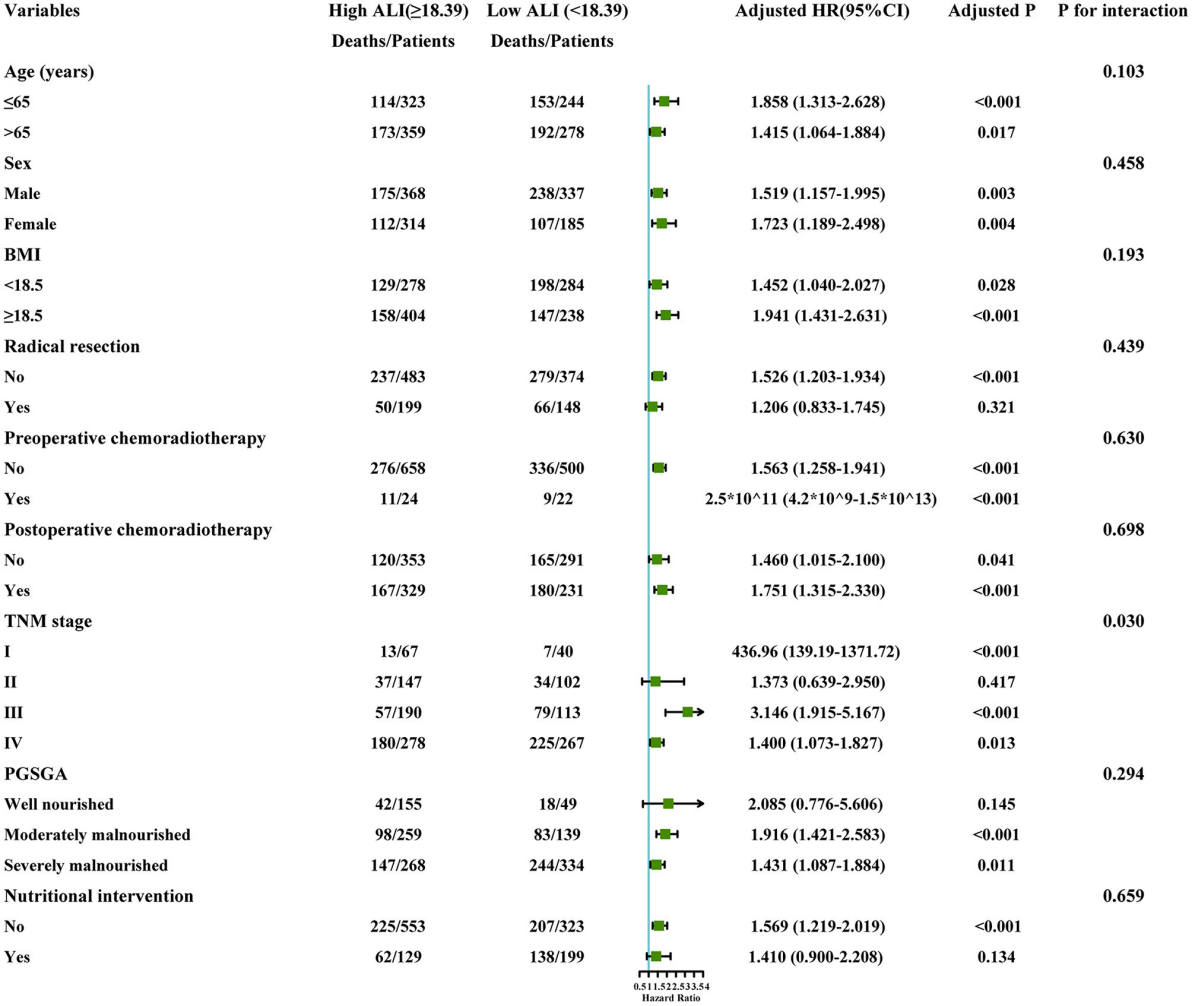
The stratification analysis of the ALI. Adjusted for model 2: Age, Sex, Radical resection, TNM stage, EORTC QLQ-C30, KPS, Neoadjuvant chemoradiotherapy, Post-operative chemoradiotherapy, Lymphocytes, Neutrophils, WBC, AST, ALT, Serum albumin, Comorbid disease (s), Family history of cancer, Tea consumption, Alcohol consumption, Smoking, Platelet, Hemoglobin, Serum total protein, PGSGA, Nutritional intervention, 30-day death, HGS. ALI, Advanced Lung Cancer Inflammation Index; WBC, White Blood Cells; AST, Aspartate Aminotransferase; ALT, Alanine Transaminase; HGS, Hand grip strength; BMI, Body Mass Index; EORTC QLQ-C30, The European Organization for Research and Treatment of Cancer (EORTC), Quality of Life Questionnaire-Core 30 (QLQ-C30); PGSGA, Patient-Generated Subjective Global Assessment; KPS, Karnofsky Performance Status.

Subgroup analysis was performed to assess the association between the ALI and the risk of death in different subgroups. A significantly interactive association between the ALI (high ALI, ≥18.39 vs. low ALI, <18.39) and death risk was observed in the TNM stage (*P* for interaction = 0.030). However, no other significant association was found for the subgroup variables (*P* for interaction > 0.05) (Figure 5).

## Discussion

To our knowledge, this was the first study to investigate the combined effects of systemic inflammatory indicators and malnutrition on the prognosis of patients with cancer sarcopenia. In our study, the C-index, DCA, and prognostic AUC of the ALI in patients with cancer sarcopenia were higher or better than those of the NLR, PNI, SII, and PLR. Accordingly, we chose the ALI score as the optimal inflammation-related index for prognosis-related analysis. When we compared the ALI in patients with cancer sarcopenia and non-cancer sarcopenia, we found that the ALI in different tumor types, TNM stage, age, and sex showed that the ALI in patients with cancer sarcopenia was lower than that in patients with non-cancer sarcopenia. This further demonstrates the distinguishing ability of the ALI in patients with cancer sarcopenia. The physical function score and quality of life score of patients with high ALI were higher than those of patients with low ALI, while the symptom score showed the opposite result. In other words, patients with low ALI have a worse quality of life than patients with high ALI, and this often indicates a poor prognosis. Systemic inflammation is often activated in cancer patients and is associated with the development of anorexia, fatigue, impaired physical activity, and weight loss (16). These are well-related to the composition of the ALI, and also reflect the inflammatory directional and physical function activities of the ALI in patients with cancer sarcopenia.

The ALI is composed of BMI, albumin, and NLR, which can reflect the inflammatory status of the host (17, 18). In previous studies, BMI was reported to be associated with skeletal sarcopenia, which is an important component of cancer cachexia syndrome and an important prognostic factor for patients with cancer (19). Additionally, serum albumin levels were affected by the SIR^12^. A study by Evans et al. recommended that an abnormal serum albumin should be considered a chronic disease characterized by inflammation and correlates well with the risk of adverse patient outcomes, and the serum albumin concentration decreased when inflammation was present (20). Inflammation is involved in carcinogenesis and cancer development (21), and SIR is considered the seventh hallmark of cancer through host tumor interaction (22). Consistent with this evidence, the potential of the SIR status as a prognostic marker of various cancers has also been confirmed, and the NLR is a reliable SIR marker. The NLR is composed of the neutrophil and lymphocyte counts. The tumor microenvironment is rich in neutrophils. The role of neutrophils in promoting inflammation and providing an appropriate environment for tumor growth explains that neutrophils activate various inflammatory markers, such as vascular endothelial growth factor and anti-apoptosis factors, such as the nuclear factor kappa light chain enhancer of activated B cells, promoting extracellular matrix remodeling and tumor progression (21, 23).

In contrast, the lymphocyte count reflects the activation of the immune system and its inhibitory effect on tumor proliferation and migration (17). A lower ALI score is associated with decreased BMI and serum albumin levels accompanied by increase in the NLR levels, representing a higher level of inflammation. In our baseline data analysis, we also found that the BMI and serum albumin levels of patients with a low ALI were lower than those of patients with a high ALI. As cancer sarcopenia results from chronic systemic inflammation, the combination of BMI, serum albumin, and inflammation markers (NLR) can more accurately assess cancer sarcopenia. In addition, the ALI seems to have better prognostic value in advanced stages of cancer (24). Our baseline data also showed that most patients with cancer sarcopenia were at an advanced stage. Therefore, the ALI has excellent prognostic value for patients with cancer sarcopenia.

When analyzing the prognostic value of the ALI, it was found that the risk of death increased with decrease in the ALI. The ALI score is an independent prognostic factor for patients with cancer. Tumor stage was closely related to the ALI. It has been reported that the degree of systemic inflammation is related to tumor progression. However, even at the same stage, the degree of inflammation may vary depending on the type of cancer (25). Additionally, we also found that the co-occurrence of a low ALI and severe malnutrition was associated with 2.262-fold mortality risk among patients with cancer sarcopenia compared with those with high ALI who were well-nourished. In malignant tumors, the systemic inflammatory response and nutritional status are both definite prognostic factors. Increasing evidence has shown that SIR was closely related to the nutritional status of various types of cancer (26). The ALI is a new malignant tumor index recently described, and the potential of the ALI as a prognostic factor for various types of cancer has gradually been revealed, such as for lung cancer (27, 28), gastric cancer (18, 29), colorectal cancer (25, 30), pancreatic cancer (31, 32), esophageal cancer (33), head and neck squamous cell carcinoma (34), nasopharyngeal carcinoma (35), thymic epithelial tumors (36), and melanoma (37). We hypothesize that the systemic inflammation reflected by the ALI is the basis of sarcopenia. The cytokine concentration in the inflammatory environment increases. The cytokines secreted by the tumor and surrounding cells can promote protein degradation (38), inhibit the differentiation of skeletal muscle cells, promote muscle wasting (39), and promote insulin resistance (40).

Increased cytokine concentrations in the circulation can activate the ubiquitin-proteasome proteolytic pathway, leading to insulin resistance and muscle wasting, thereby further aggravating sarcopenia (41). On the other hand, local muscle inflammation can further promote systemic inflammation and muscle interpretation (6, 7). Skeletal sarcopenia may also be caused by malnutrition (42). Nutritional and metabolic disorders are very common in patients with advanced cancer and can lead to weight loss, reduced quality of life, and poor treatment outcomes (43). The degree of malnutrition is affected by several factors, including anorexia and reduced nutritional intake (44). Insufficient energy and protein intake were independent risk factors for skeletal sarcopenia (42). A poor nutritional status can lead to immune dysfunction and muscle atrophy (45). Systemic inflammation is related to anorexia and insufficient nutrient intake, which in turn lead to accelerated loss of skeletal muscle. In some patients, inflammation causes anorexia and is accompanied by decrease in skeletal muscle (4). Malnutrition can also impair the immune response and damage host defenses against cancer (46). In short, systemic inflammation, malnutrition and sarcopenia are closely related, forming a vicious circle. In our study, the number of malnourished patients diagnosed with the PCSGA was as high as 83.1% (including 50% of severely malnourished patients), but only 27.2% of patients received a nutritional intervention. Adequate nutrition and resistance exercise are the basis for the management of sarcopenia, and multimodal interventions are often associated with the best outcomes. Systemic inflammation and malnutrition are problems that patients with cancer cannot avoid. Therefore, strengthening the comprehensive treatment of patients to prevent muscle consumption and improve physical condition, strength and quality of life is urgently needed (47).

To our knowledge, this was the first study to investigate the combined effects of systemic inflammation and malnutrition in patients with cancer. Although ours was a multicenter cohort study of patients with cancer sarcopenia, we acknowledge some potential limitations. Regardless of the systemic inflammation indicators examined and the subgroups defined according to age, BMI, sex, and tumor stage, the results were basically the same. Other indicators of inflammation, such as interleukin-6, TNF-α and CRP should be collected in our cohort in the future. Notably, a study with a larger sample size and more participating centers is needed to verify the conclusions. In addition, it is imperative to conduct prospective clinical trials of comprehensive treatment, including anti-inflammatory and nutritional interventions.

## Conclusion

In summary, our study found that the ability of the ALI to distinguish and predict the prognosis of patients with cancer sarcopenia was better than that of the NLR, PNI, SII, and PLR. Low ALI levels are associated with a worse prognosis in patients with cancer and sarcopenia. We also found that patients with both a low ALI who were severe malnutrition had a nearly three-fold higher risk of mortality compared to patients with a high ALI and well-nourished. Systemic inflammation, malnutrition, and sarcopenia affect each other. For patients with cancer sarcopenia, it is necessary to develop comprehensive treatment with the aim of reducing systemic inflammation, strengthening nutritional intervention, and improving skeletal muscle mass.

## Data Availability Statement

The datasets used and analyzed during the current study are available from the corresponding author on reasonable request.

## Ethics Statement

This study followed the tenets of the Helsinki declaration. All participants signed an informed consent form and this study was approved by the Institutional Review Board of each hospital (Registration Number: ChiCTR1800020329).

## Author Contributions

G-TR wrote the manuscript. G-TR, Y-ZG, and H-LX analyzed and interpreted the patient data. G-TR, Y-ZG, H-LX, and H-PS made substantial contributions to the conception, design, and intellectual content of the studies. All authors read and approved the final manuscript.

## Funding

This work was supported by the National Key Research and Development Program (Grant Number 2017YFC1309200).

## Conflict of Interest

The authors declare that the research was conducted in the absence of any commercial or financial relationships that could be construed as a potential conflict of interest.

## Publisher's Note

All claims expressed in this article are solely those of the authors and do not necessarily represent those of their affiliated organizations, or those of the publisher, the editors and the reviewers. Any product that may be evaluated in this article, or claim that may be made by its manufacturer, is not guaranteed or endorsed by the publisher.

## Supplementary Material

Supplementary Figure S1 Optimal cut-off value of inflammation markers according to the results of the standardized log-rank statistic.

Supplementary Figure S2 Flowchart of patient selection for this study.

Supplementary Figure S3 The 1-, 3-, and 5-year calibration curves of the ALI in patients with cancer sarcopenia. ALI, advanced lung cancer inflammation index.

Supplementary Figure S4 The Kaplan-Meier survival curves of ALI and PGSGA in the OS of patients with sarcopenia. ALI, advanced lung cancer inflammation index; OS, overall survival; PG-SGA, Patient-Generated Subjective Global Assessment.

Supplementary Table S1 Demographic and clinical characteristics stratified by ALI.

Supplementary Table S2 The EORTC QLQ-C30 of overall patients and different ALI group.

Supplementary Table S3 Sensitivity analysis of the OS in patients with cancer sarcopenia.

Supplementary Table S4 Univariate and multivariate analysis of the OS in patients with cancer sarcopenia.
