# Supplementary figures and images for: Association Between Systemic Inflammation and Malnutrition With Survival in Patients With Cancer Sarcopenia—A Prospective Multicenter Study
Source: Front Nutr. 2022 Feb 7;8:811288. doi: 10.3389/fnut.2021.811288 (PMC8859438; doi:10.3389/fnut.2021.811288)

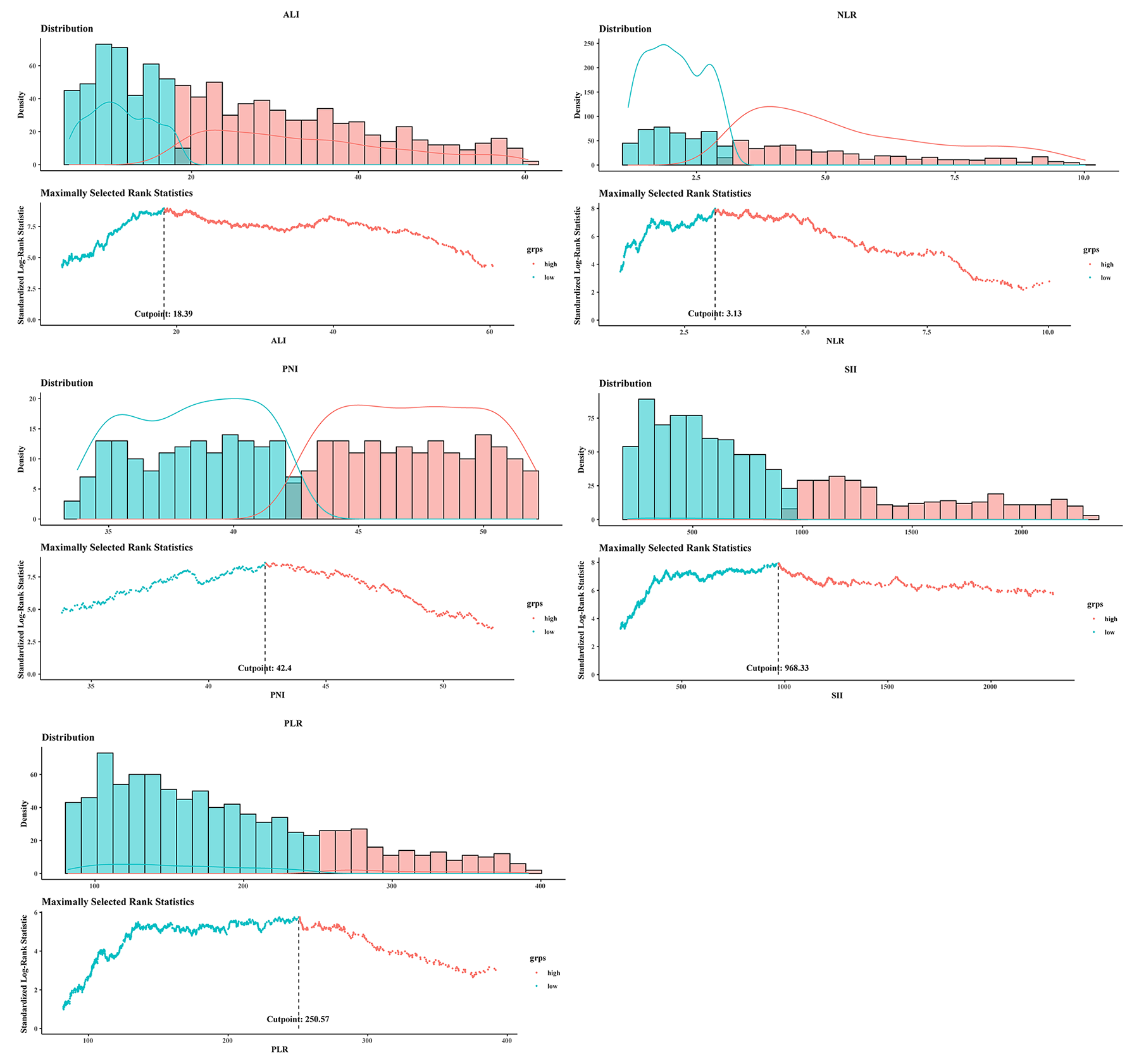

Supplement: Supplementary Figure S1 — Optimal cut-off value of inflammation markers according to the results of the standardized log-rank statistic. [file Image_1.TIF]

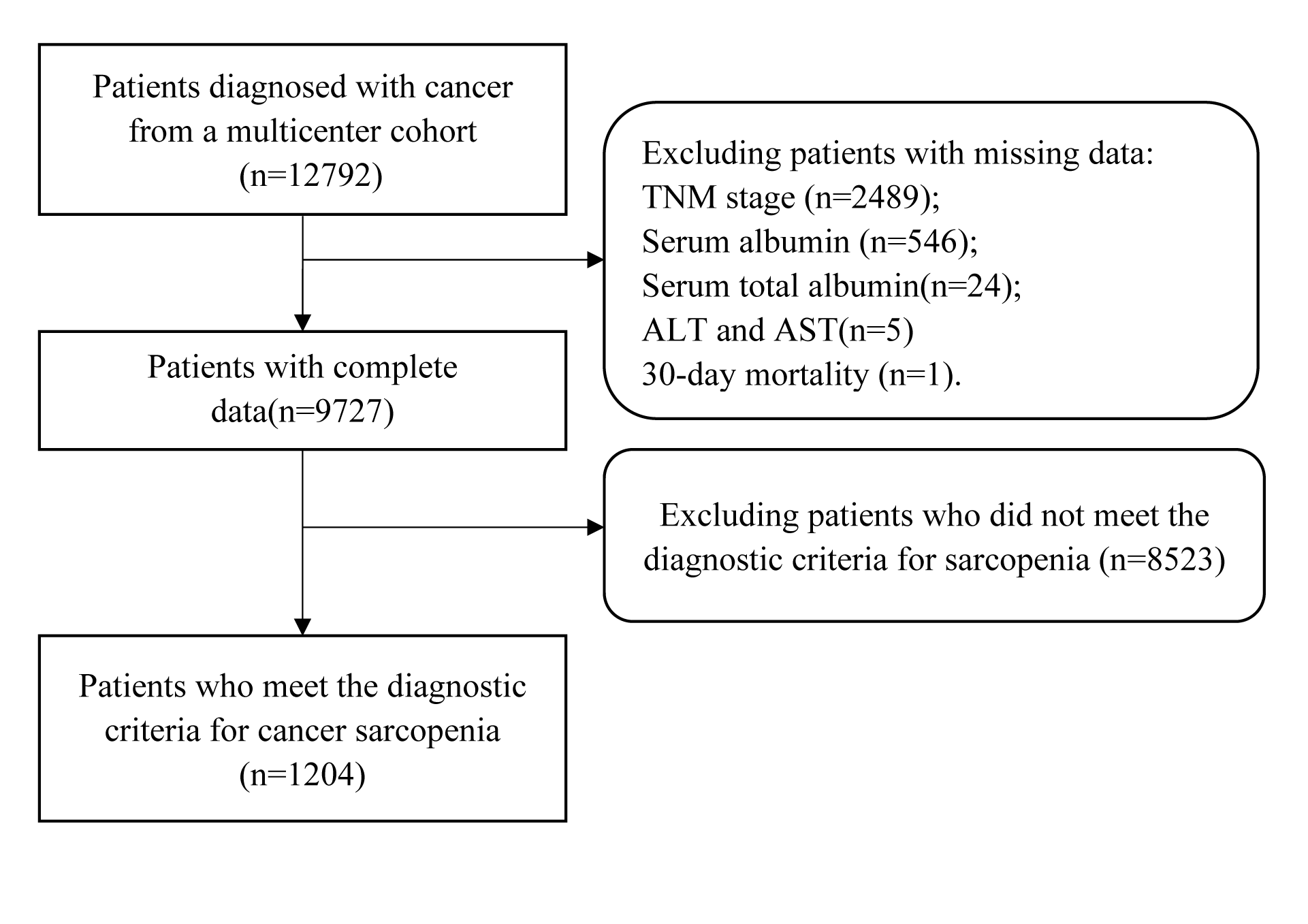

Supplement: Supplementary Figure S2 — Flowchart of patient selection for this study. [file Image_2.TIF]

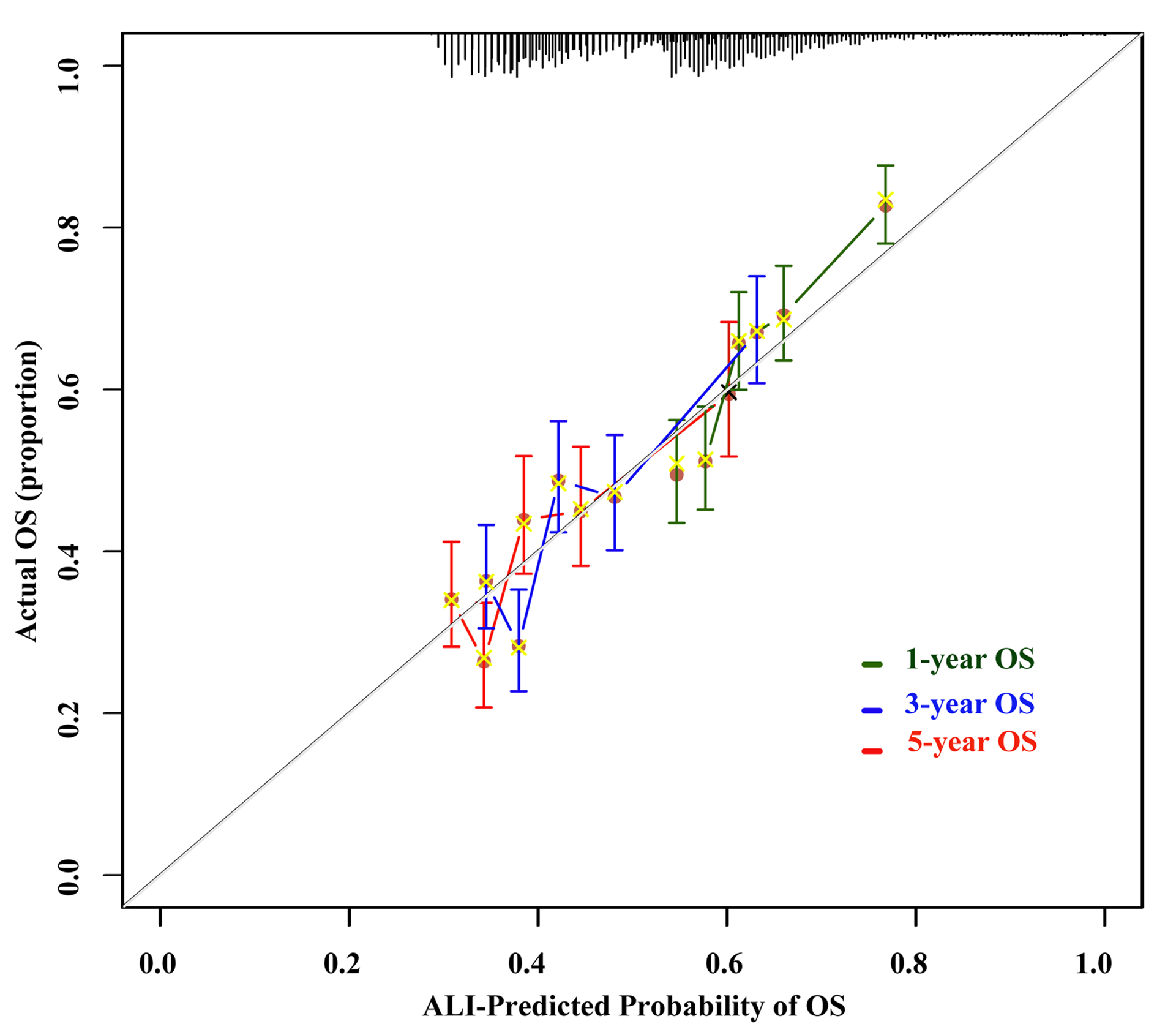

Supplement: Supplementary Figure S3 — The 1-, 3-, and 5-year calibration curves of the ALI in patients with cancer sarcopenia. ALI, advanced lung cancer inflammation index. [file Image_3.TIF]

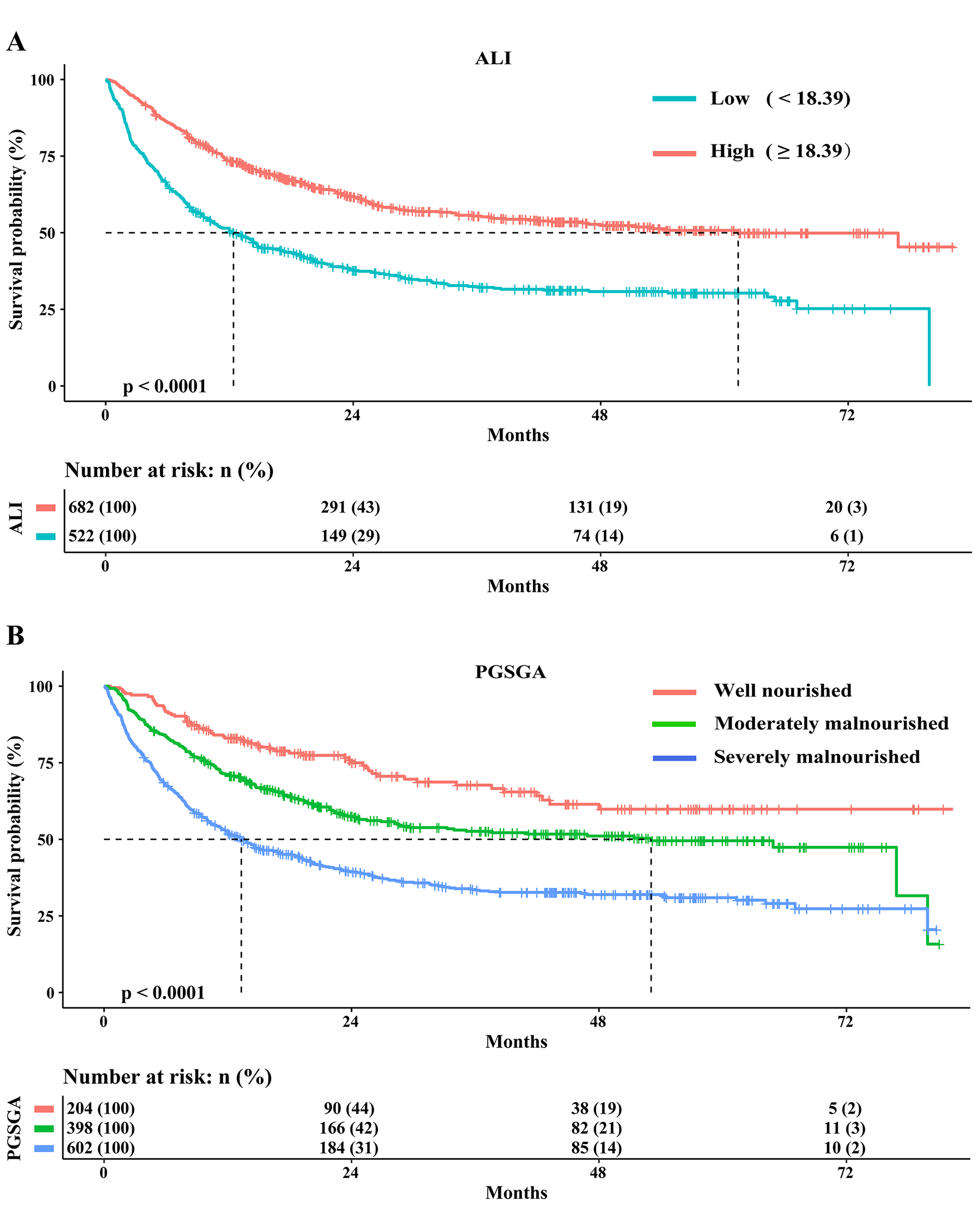

Supplement: Supplementary Figure S4 — The Kaplan-Meier survival curves of ALI and PGSGA in the OS of patients with sarcopenia. ALI, advanced lung cancer inflammation index; OS, overall survival; PG-SGA, Patient-Generated Subjective Global Assessment. [file Image_4.TIF]
