# Supplementary material for: Association Between Systemic Inflammation and Malnutrition With Survival in Patients With Cancer Sarcopenia—A Prospective Multicenter Study
Source: Front Nutr. 2022 Feb 7;8:811288. doi: 10.3389/fnut.2021.811288 (PMC8859438; doi:10.3389/fnut.2021.811288)
Supplement: Supplementary Table S1 — Demographic and clinical characteristics stratified by ALI. [file Table_1.DOCX]

**Table S1 Demographic and clinical characteristics stratified by ALI**

|  | Stratified by ALI | |  |
| --- | --- | --- | --- |
| Characteristics | High ALI (≥18.39) | High ALI (<18.39) | *P* value |
|  | (n=682) | (n=522) |  |
| Age, years, (mean (SD)) | 64.55 (11.23) | 64.46 (11.67) | 0.884 |
| Sex, n (%) |  |  | <0.001 |
| Male | 368 (54.00) | 337 (64.60) |  |
| Female | 314 (46.00) | 185 (35.40) |  |
| Sites of cancer, n (%) |  |  | <0.001 |
| Lung cancer, n (%) | 120 (17.60) | 119 (22.80) |  |
| Gastric cancer, n (%) | 143 (21.00) | 102 (19.50) |  |
| Colorectal cancer, n (%) | 144 (21.10) | 126 (24.10) |  |
| Esophageal cancer, n (%) | 90 (13.20) | 55 (10.50) |  |
| Hepatobiliary cancer, n (%) | 11 (1.60) | 20 (3.80) |  |
| Pancreatic cancer, n (%) | 23 (3.40) | 19 (3.60) |  |
| Breast cancer, n (%) | 50 (7.30) | 14 (2.70) |  |
| Utero ovarian cancer, n (%) | 33 (4.80) | 32 (6.10) |  |
| Nasopharyngeal cancer, n (%) | 39 (5.70) | 16 (3.10) |  |
| Urological cancer, n (%) | 10 (1.50) | 7 (1.30) |  |
| Other cancer subtypes, n (%) | 19 (2.80) | 12 (2.30) |  |
| Comorbid disease(s), yes, n (%) |  |  | 0.637 |
| 0 | 436 (63.90) | 319 (61.10) |  |
| 1 | 179 (26.20) | 141 (27.00) |  |
| 2 | 46 (6.70) | 41 (7.90) |  |
| 3 or more | 21 (3.10) | 21 (4.00) |  |
| Family history of cancer, yes, n (%) | 91 (13.30) | 68 (13.00) | 0.940 |
| Smoking, yes, n (%) | 309 (45.30) | 263 (50.40) | 0.091 |
| Alcohol consumption, yes, n (%) | 127 (18.60) | 115 (22.00) | 0.164 |
| Tea consumption, n (%) | 152 (22.30) | 128 (24.50) | 0.401 |
| BMI, kg/m^2 (mean (SD)) | 18.68 (1.70) | 18.08 (1.82) | <0.001 |
| TNM stage, n (%) |  |  | 0.003 |
| Ⅰ | 67 (9.80) | 40 (7.70) |  |
| Ⅱ | 147 (21.60) | 102 (19.50) |  |
| Ⅲ | 190 (27.90) | 113 (21.60) |  |
| Ⅳ | 278 (40.80) | 267 (51.10) |  |
| Radical resection, yes, n (%) | 199 (29.20) | 148 (28.40) | 0.803 |
| Neoadjuvant chemoradiotherapy, yes, n (%) | 24 (3.50) | 22 (4.20) | 0.637 |
| Postoperative chemoradiotherapy, yes, n (%) | 329 (48.20) | 231 (44.30) | 0.188 |
| EORTC QLQ-C30 | 51.14 (8.58) | 48.04 (9.85) | <0.001 |
| KPS (mean (SD)) | 83.99 (13.63） | 74.67 (20.14） | <0.001 |
| Serum total protein (g/L) (mean (SD)) | 67.72 (7.20) | 62.14 (8.33) | <0.001 |
| Serum albumin (g/L) (mean (SD)) | 38.50 (4.80) | 33.42 (5.69) | <0.001 |
| AST (U/L) (median (IQR)) | 21.20 (17.00-27.85) | 22.15(16.20,33.00) | <0.001 |
| ALT (U/L) (median (IQR)) | 16.00 (11.43, 23.73) | 17.65 (11.00, 30.23) | <0.001 |
| Hemoglobin (g/L) (mean (SD)) | 118.38 (19.47） | 106.79 (21.60） | <0.001 |
| WBC (×10^9/L) (mean (SD)) | 5.63 (2.02) | 8.91 (4.53) | <0.001 |
| Neutrophils (×10^9/L) (mean (SD)) | 3.27 (1.59) | 7.08 (4.24) | <0.001 |
| Lymphocytes (×10^9/L) (mean (SD)) | 1.72 (0.95) | 1.01 (0.51) | <0.001 |
| Platelet (×10^9/L) (mean (SD)) | 223.75 (88.51） | 241.27 (108.35） | 0.002 |
| 30-day death, yes, n (%) | 4 (0.60) | 28 (5.40) | <0.001 |
| PGSGA, n (%) |  |  | <0.001 |
| Well nourished | 155 (22.70) | 49 (9.40) |  |
| Moderately malnourished | 259 (38.00) | 139 (26.60) |  |
| Severely malnourished | 268 (39.30) | 334 (64.00) |  |
| Nutritional intervention, yes, n (%) | 129 (18.90) | 199 (38.10) | 0.163 |
| HGS (mean (SD)), (Kg) | 16.77 (6.20) | 16.25 (6.670) |  |

Notes: BMI: Body Mass Index; EORTC QLQ-C30, The European Organization for Research and Treatment of Cancer (EORTC), Quality of Life Questionnaire-Core 30 (QLQ-C30); KPS, Karnofsky Performance Status; AST: Aspartate Aminotransferase; ALT: Alanine Transaminase; WBC: White Blood Cells; ALI: Advanced Lung Cancer Inflammation Index; NLR: Neutrophil-Lymphocyte Ratio; PNI: Prognostic Nutritional Index; SII: Systemic Immune-Inflammation Index; PLR: Platelet-Lymphocyte Ratio; PGSGA: Patient-Generated Subjective Global Assessment.
