# Supplementary material for: Association Between Systemic Inflammation and Malnutrition With Survival in Patients With Cancer Sarcopenia—A Prospective Multicenter Study
Source: Front Nutr. 2022 Feb 7;8:811288. doi: 10.3389/fnut.2021.811288 (PMC8859438; doi:10.3389/fnut.2021.811288)
Supplement: Supplementary Table S2 — The EORTC QLQ-C30 of overall patients and different ALI group. [file Table_2.DOCX]

**Table S2 The EORTC QLQ-C30 of overall patients and different ALI group**

| Characteristic | Overall  n= 1204 | Low ALI  n= 522 | High ALI  n= 682 | *p*-value |
| --- | --- | --- | --- | --- |
| Physical function | 86.67 (26.66) | 73.33 (40.00) | 86.67 (26.67) | <0.001 |
| Role function | 66.67 (33.33) | 66.67 (66.67) | 75.00 (33.33) | <0.001 |
| Emotional function | 91.67 (25.00) | 91.67 (33.33) | 100.00 (25.00) | <0.001 |
| Cognitive function | 83.33 (33.33) | 83.33 (33.33) | 100.00 (16.67) | 0.001 |
| Social function | 66.67 (33.33) | 66.67 (66.67) | 66.67 (33.33) | <0.001 |
| Global QOL | 50.00 (16.67) | 50.00 (33.34) | 58.33(16.67) | <0.001 |
| Fatigue | 22.22 (33.33) | 33.33 (33.33) | 22.22 (33.30) | <0.001 |
| Nausea and vomiting | 0.00 (0.00) | 0.00 (16.70) | 0.00 (0.00) | <0.001 |
| Pain | 0.00 (33.30) | 16.70 (33.33) | 0.00 (0.00) | <0.001 |
| Dyspnea | 0.00 (33.30) | 0.00 (33.33) | 0.00 (33.33) | <0.001 |
| Sleep disturbance | 0.00 (33.30) | 33.30 (33.33) | 33.30 (33.30) | 0.001 |
| Appetite loss | 0.00 (33.30) | 33.30 (33.30) | 33.30 (33.30) | <0.001 |
| Constipation | 0.00 (0.00) | 0.00 (0.00) | 0.00 (33.30) | 0.277 |
| Diarrhea | 0.00 (0.00) | 0.00 (0.00) | 0.00 (0.00) | 0.018 |
| Financial difficulties | 33.30 (66.67) | 33.3 (66.67) | 33.30 (66.67) | 0.001 |
| Function score (1-5) | 80.83(24.34) | 76.67(29.50) | 83.50 (20.91) | <0.001 |
| Symptom score (7-9) | 14.82(17.28) | 18.52(28.40) | 11.73(15.43) | <0.001 |

Notes: Data are represented as median (interquartile range).
