# Supplementary material for: Association Between Systemic Inflammation and Malnutrition With Survival in Patients With Cancer Sarcopenia—A Prospective Multicenter Study
Source: Front Nutr. 2022 Feb 7;8:811288. doi: 10.3389/fnut.2021.811288 (PMC8859438; doi:10.3389/fnut.2021.811288)
Supplement: Supplementary Table S3 — Sensitivity analysis of the OS in patients with cancer sarcopenia. [file Table_3.docx]

**Table S3 Sensitivity analysis of the OS in patients with cancer sarcopenia**

| Variables | OS (model 0) | |  | OS (model 1) | |  | OS (model 2) | |  |
| --- | --- | --- | --- | --- | --- | --- | --- | --- | --- |
|  | Crude HR (95%CI) | Crude *P* |  | Adjusted HR (95%CI) | Adjusted *P* | | Adjusted HR (95%CI) | Adjusted *P* | |
| ALI* |  |  |  |  |  |  |  |  |  |
| As continuous | 0.706 (0.464-1.074) | 0.104 |  | 0.808 (0.559-1.168) | 0.256 |  | 0.860 (0.590-1.255) | 0.436 |  |
| By cut-off |  |  |  |  |  |  |  |  |  |
| ALI≥18.39 | 1 |  |  | 1 |  |  | 1 |  |  |
| ALI <18.39 | 1.588 (1.288-1.958) | <0.001 |  | 1.687 (1.365-2.085) | <0.001 |  | 2.026 (1.53-2.683) | <0.001 |  |
| By Interquartile |  |  |  |  |  |  |  |  |  |
| Q1 (37.94~) | 1 |  |  | 1 |  |  | 1 |  |  |
| Q2 (21.26~37.94) | 1.417 (1.045-1.921) | 0.025 |  | 1.292 (0.952-1.754) | 0.100 |  | 1.292 (0.940-1.778) | 0.015 |  |
| Q3 (10.84~21.26) | 2.105 (1.570-2.822) | <0.001 |  | 1.956 (1.456-2.628) | <0.001 |  | 2.130 (1.516-2.992) | <0.001 |  |
| Q4 (~10.84) | 1.612 (1.179-2.204) | 0.003 |  | 1.750 (1.276-2.401) | <0.001 |  | 2.617 (1.649-4.152) | <0.001 |  |
| *P* for trends |  | <0.001 |  |  |  |  |  | <0.001 |  |
| ALI**^#^** |  |  |  |  |  |  |  |  |  |
| As continuous | 0.690 (0.560-0.849) | <0.001 |  | 0.692 (0.558-0.857) | <0.001 |  | 0.796（0.631-1.001） | 0.055 |  |
| By cut-off |  |  |  |  |  |  |  |  |  |
| ALI≥18.39 | 1 |  |  | 1 |  |  | 1 |  |  |
| ALI <18.39 | 1.998 (1.539-2.594) | <0.001 |  | 2.175 (1.480-3.195) | <0.001 |  | 2.376 (1.636-3.451) | <0.001 |  |
| By Interquartile |  |  |  |  |  |  |  |  |  |
| Q1 (37.94~) | 1 |  |  | 1 |  |  | 1 |  |  |
| Q2 (21.26~37.94) | 1.444 (0.961-2.170) | 0.077 |  | 1.410 (0.936-2.122) | 0.100 |  | 1.445 (0.940-2.220) | 0.093 |  |
| Q3 (10.84~21.26) | 2.431 (1.668-3.544) | <0.001 |  | 2.446 (1.673-3.577) | <0.001 |  | 2.666 (1.690-4.207) | <0.001 |  |
| Q4 (~10.84) | 2.155 (1.475-3.149) | <0.001 |  | 2.175 (1.480-3.195) | <0.001 |  | 2.508 (1.372-4.583) | 0.003 |  |
| *P* for trends |  | <0.001 |  |  | <0.001 |  |  | <0.001 |  |

Notes: ALI*: The sensitivity analysis was to exclude patients who died within 6 months. ALI**^#^**: The sensitivity analysis was to exclude patients who TNM stage was IV. Model 0: Unadjusted; Model 1: Adjusted for Age, Sex and TNM stage; Model 2: Adjusted for Age, Sex, Radical resection, TNM stage, EORTC QLQ-C30, KPS, Neoadjuvant chemoradiotherapy, Postoperative chemoradiotherapy, Lymphocytes, Neutrophils, WBC, AST, ALT, Serum albumin, Comorbid disease(s), Family history of cancer, Tea consumption, Alcohol consumption, Smoking, Platelet, Hemoglobin, Serum total protein, PGSGA, Nutritional intervention, 30-day mortality, HGS, and tumor types. OS, Overall Survival; HR, Hazards Ratio; CI, Confidence Interval; BMI: Body Mass Index; EORTC QLQ-C30, The European Organization for Reasearch and Treatment of Cancer (EORTC), Quality of Life Questionnaire-Core 30 (QLQ-C30); KPS, Karnofsky Performance Status; AST: Aspartate Aminotransferase; ALT: Alanine Transaminase; WBC: White Blood Cells; HGS: Hand grip strength; ALI: Advanced Lung Cancer Inflammation Index; PGSGA: Patient-Generated Subjective Global Assessment.
