# Supplementary material for: Association Between Systemic Inflammation and Malnutrition With Survival in Patients With Cancer Sarcopenia—A Prospective Multicenter Study
Source: Front Nutr. 2022 Feb 7;8:811288. doi: 10.3389/fnut.2021.811288 (PMC8859438; doi:10.3389/fnut.2021.811288)
Supplement: Supplementary Table S4 — Univariate and multivariate analysis of the OS in patients with cancer sarcopenia. [file Table_4.docx]

**Table S4 Univariate and multivariate analysis of the OS in patients with cancer sarcopenia**

| Variables | OS | |  | OS * | | |  |
| --- | --- | --- | --- | --- | --- | --- | --- |
|  | Crude HR (95%CI) | Crude *P* | Adjusted HR (95%CI) | | Adjusted *P* | | |
| Lung cancer |  |  |  |  | |  |  |
| ALI≥18.39 | 1 |  |  | 1 | |  |  |
| ALI <18.39 | 1.974 (1.46-2.668) | <0.001 |  | 1.119 (0.723-1.732) | | 0.641 |  |
| Gastrointestinal cancer |  |  |  |  | |  |  |
| Gastric cancer |  |  |  |  | |  |  |
| ALI≥18.39 | 1 |  |  | 1 | |  |  |
| ALI <18.39 | 1.553 (1.108-2.177) | 0.011 |  | 1.612 (0.982-2.647) | | 0.059 |  |
| Colorectal cancer |  |  |  |  | |  |  |
| ALI≥18.39 | 1 |  |  | 1 | |  |  |
| ALI <18.39 | 1.941 (1.342-2.808) | <0.001 |  | 2.347 (1.286-4.284) | | 0.005 |  |
| Esophageal cancer |  |  |  |  | |  |  |
| ALI≥18.39 | 1 |  |  | 1 | |  |  |
| ALI <18.39 | 2.505 (1.626-3.860) | <0.001 |  | 2.099 (0.918-4.799) | | 0.079 |  |
| Hepatobiliary cancer |  |  |  |  | |  |  |
| ALI≥18.39 | 1 |  |  | 1 | |  |  |
| ALI <18.39 | 1.115 (0.653-1.903) | 0.691 |  | 0.592 (0.174-2.019) | | 0.403 |  |
| Other cancer subtypes |  |  |  |  | |  |  |
| ALI≥18.39 | 1 |  |  | 1 | |  |  |
| ALI <18.39 | 4.002 (2.438-6.572) | <0.001 |  | 7.108 (3.023-16.717) | | <0.001 |  |

Notes: OS, Overall Survival; HR, Hazards Ratio; CI, Confidence Interval; BMI: Body Mass Index; EORTC QLQ-C30, The European Organization for Research and Treatment of Cancer (EORTC), Quality of Life Questionnaire-Core 30 (QLQ-C30); KPS, Karnofsky Performance Status; AST: Aspartate Aminotransferase; ALT: Alanine Transaminase; WBC: White Blood Cells; HGS: Hand grip strength; ALI: Advanced Lung Cancer Inflammation Index; PGSGA: Patient-Generated Subjective Global Assessment.

*: Adjusted for Age, Sex, Radical resection, TNM stage, EORTC QLQ-C30, KPS, Neoadjuvant chemoradiotherapy, Postoperative chemoradiotherapy, Lymphocytes, Neutrophils, WBC, AST, ALT, Serum albumin, Comorbid disease(s), Family history of cancer, Tea consumption, Alcohol consumption, Smoking, Platelet, Hemoglobin, Serum total protein, PGSGA, Nutritional intervention, 30-day mortality, and HGS.
